# The Predictive Role of Neutrophil-to-Lymphocyte Ratio (NLR), Platelet-to-Lymphocyte Ratio (PLR), Monocytes-to-Lymphocyte Ratio (MLR) and Gammaglobulins for the Development of Cutaneous Vasculitis Lesions in Primary Sjögren’s Syndrome

**DOI:** 10.3390/jcm11195525

**Published:** 2022-09-21

**Authors:** Ancuta Mihai, Ana Caruntu, Daniela Opris-Belinski, Ciprian Jurcut, Alina Dima, Constantin Caruntu, Ruxandra Ionescu

**Affiliations:** 1 Department of Internal Medicine, Carol Davila Central Military Emergency Hospital, 010825 Bucharest, Romania; 2 Department of Rheumatology, Faculty of Medicine, Titu Maiorescu University, 031593 Bucharest, Romania; 3 Department of Oral and Maxillofacial Surgery, Carol Davila Central Military Emergency Hospital, 010825 Bucharest, Romania; 4 Department of Oral and Maxillofacial Surgery, Faculty of Dental Medicine, Titu Maiorescu University, 031593 Bucharest, Romania; 5 Department of Internal Medicine and Rheumatology, Sfanta Maria Clinical Hospital, 011172 Bucharest, Romania; 6 Department of Internal Medicine and Rheumatology, Carol Davila University of Medicine and Pharmacy, 020021 Bucharest, Romania; 7 Department of Rheumatology, Colentina Clinical Hospital, 020125 Bucharest, Romania; 8 Department of Physiology, Carol Davila University of Medicine and Pharmacy, 020021 Bucharest, Romania; 9 Department of Dermatology, Prof. N.C. Paulescu National Institute of Diabetes, Nutrition and Metabolic Diseases, 011233 Bucharest, Romania

**Keywords:** primary Sjögren’s syndrome, neutrophil to lymphocyte ratio, platelet to lymphocyte ratio, monocytes to lymphocyte ratio, gammaglobulins, cutaneous vasculitis lesions, predictors

## Abstract

Background: In primary Sjögren’s Syndrome (pSS), cutaneous vasculitis lesions (CVL) are extraglandular manifestations with an important clinical and prognostic impact and their early detection might contribute to the improvement of disease control and even patients’ survival. The aim of this study was to evaluate the predictive potential of hematological elements in the development of CVL in pSS patients. Methods: In this single center, retrospective study, a total of 245 participants were included (124 pSS patients and 121 healthy controls). Complete blood count, inflammatory and immunological parameters were determined at the initial visit. pSS patients underwent a periodical follow-up program, when disease progression and response to therapy was monitored, including the emergence of CVL. Results: In pSS, leucocytes, lymphocyte, neutrophil, monocyte, erythrocyte and platelet counts are significantly decreased compared to healthy subjects (*p* < 0.001), whereas cellular ratios: NLR, PLR, MLR, and immunological and inflammatory parameters are significantly increased (*p* < 0.001). A total of 34 patients with pSS (27.41%) developed CVL during the follow-up period. The occurrence of CVL was positively correlated with neutrophil and platelet counts (*p* < 0.001), while for lymphocytes the correlation was negative (*p* < 0.001). Cellular ratios: NLR, PLR and MLR, and gammaglobulins also revealed significant positive correlations with the emergence of CVL in pSS (*p* < 0.001). The multivariate analysis confirmed the independent predictive character for CVL emergence in pSS for NLR (CI95% 0.053–0.2, *p* < 0.002), PLR (CI95% 0.001–0.003, *p* < 0.003), MLR (CI95% 0.086–0.935, *p* < 0.019), and gammaglobulins (CI95% 0.423–0.688, *p* < 0.001). Conclusions: Standard hematological parameters, widely used in the assessment of pSS patients, such as NLR, PLR, MLR and gammaglobulins could become valid elements that might be used for the early detection of patients at risk for the development of CVL.

## 1. Introduction

Primary Sjögren’s syndrome (pSS) is a chronic autoimmune rheumatic disease, characterized by dryness of eyes and mouth due to lymphocytic infiltration of the lacrimal and salivary glandular tissues, accompanied by polyclonal or oligoclonal B cell activation and production of autoantibodies [1]. The incidence of pSS is between 3.9 and 5.3 cases per 100.000 patients/year, with an estimated prevalence between 0.2 and 2.7%. It has a female to male ratio of 9:1 and dual-mode age distribution, with a first peak in the fourth decade of life and a second peak in the fifth decade, while being rare in children [2].

Exocrine glands are the primary affected tissue in pSS, but almost any organ can be involved. Therefore, Sjögren’s syndrome can show a broad clinical spectrum and a variety of serious complications such as polysynovitis, neuropathy, cutaneous vasculitis, tubulointerstitial nephritis, interstitial lung disease and non-Hodgkin lymphoma [3].

Cutaneous involvement in pSS is relatively common and various manifestations may be present, in particular xeroderma, eyelid dermatitis, annular erythema and non-palpable purpura [4]. Among non-vasculitis lesions, xerosis was shown to be very common in pSS, with a prevalence of up to 72% [5]. The most clinically and prognostically significant cutaneous complications associated with pSS are cutaneous vasculitis lesions (CVL). The prevalence of CVL in pSS has been reported to be 1–30.6% [5], with a predominance in female patients. In terms of outcomes, CVL represent one of the most important extra-glandular manifestations in pSS, as it is associated with more severe disease and systemic manifestations [4]. Different studies have shown that its presence, especially associated with cryoglobulinemia at the time of diagnosis, carries a higher risk of lymphoma development, and have demonstrated that patients with this complication have higher morbidity and mortality, and more frequent hospitalizations than pSS patients without CVL [6,7].

The importance of an accurate and early diagnosis in pSS patients, associated with a systematic follow-up are crucial, especially in identifying the extra-glandular manifestations, knowing that pSS remains one of the most difficult to manage chronic autoimmune disorders [8]. Moreover, the desire to identify biological parameters that can bring clinical, diagnostic and prognostic information is growing with every study.

In recent years, there has been a surge of interest in the role of hematological indicators in the assessment of autoimmune diseases activity, such as pSS, SLE, Behçet disease, systemic vasculitis and ulcerative colitis, as well as in cancer and infectious diseases [9,10,11]. Blood cells such as neutrophils, lymphocytes, platelets and monocytes play major roles in inflammatory processes [12,13]. Their ratios, including neutrophil to lymphocyte ratio (NLR), platelet to lymphocyte ratio (PLT), monocytes to lymphocyte ratio (MLR), or hematological indices like the mean platelet volume to Plateletcrit ratio (MPVPCTR) and platelet distribution width to Plateletcrit ratio (PDWPCTR), were studied in a large spectrum of diseases, including autoimmune diseases [12,13,14,15]. NLR, an index of systemic inflammation, has been identified as a useful tool for the differential diagnosis or prognosis in different diseases [14]. Moreover, recent studies highlighted that increased NLR values have been correlated with intense disease activity in autoimmune diseases [12,15]. Similar to NLR, PLR is another marker of systemic inflammation, used for the differential diagnosis or prognosis in cancers and inflammatory autoimmune diseases [16,17]. MLR is used as an index for the differential diagnosis or prognosis in cardiovascular and inflammatory diseases [18].

The aim of this study was to identify reliable and easily accessible hematological elements predictive for the development of CVL in pSS. Such elements could be used for a timely stratification of the patients into risk groups for progression to severe disease. Furthermore, individualized therapeutic approaches might be developed for high risk pSS patients from the moment of diagnosis that could minimize the risk for severe complications. 

## 2. Materials and Methods

### 2.1. Study Design and Participants

This is a retrospective study involving 245 subjects admitted to the Department of Internal Medicine, Carol Davila Central Military Emergency Hospital, between April 2015 and November 2021, of which 124 were patients diagnosed with pSS and 121 were control subjects. The diagnosis of pSS subjects was according to the American European Consensus Group criteria (AECG) [19] and ACR/EULAR classification criteria for pSS [20]. All the pSS subjects included in this study were newly diagnosed and did not receive any previous treatment for pSS. According to AECG criteria, in patients with negative serum antibodies results, biopsy of the minor salivary glands was performed, with subsequent pathology analysis of the specimen, conducted by two different experienced pathologists, confirming the diagnosis. Patients with other autoimmune diseases, such as rheumatoid arthritis, systemic lupus erythematosus, systemic vasculitis and systemic sclerosis, lymphoproliferative disorders, malignancies, liver diseases, end-stage renal disease and diabetic nephropathy, active infections, recent history of blood transfusion and anemia, were excluded. At the initial visit, a thorough general assessment was conducted, recording all glandular and extra-glandular disease expression, including cutaneous manifestations. Evaluation for skin dryness and pain was performed using a visual analogue scale (VAS) [21], with a 0–100 mm score. Blood samples were collected for analysis of complete blood count, inflammatory and immunological parameters between 7 a.m. and 11 a.m., as part of outpatient’s clinical routine. Disease activity was assessed in all patients using the ESSDAI composite score [19], a well-recognized global index calculated according to the symptoms and laboratory parameters recorded for the subjects. Considering the ESSDAI score, patients with mild disease activity (5–13 score) received conservatory treatment, represented by hygienic-dietary and topical treatments, and patients with moderate to severe disease activity (score ≥ 14) received systemic treatments. The treatment of systemic vasculitis patients was, as for other autoimmune diseases, corticosteroids, cyclophosphamide and/or azathioprine. In refractory cases, plasmapheresis and intravenous immunoglobulins (IVIgs) were effective treatments and, in cases of cryoglobulinemic vasculitis, B cell-depleting therapy with rituximab has been used. In all patients with cutaneous vasculitis lesions associated with cryoglobulinemia, the diagnosis was confirmed through biopsy.

After the initial assessment, all patients were enrolled on a periodical follow-up program, with trimestral visits, to monitor disease progression, including the occurrence of cutaneous complications and response to therapy. All patients included in the study attended a minimum of two follow-up visits.

Control subjects were selected from patients without any known diseases, who underwent routine physical examinations and general paraclinical investigations as part of the annual health control program. They were selected in the same recruiting period, based on age- and gender-matched characteristics.

The research was approved by the hospital Ethics Committee (No 365/11.02.2020) and all patients signed an informed consent at one of the periodical follow up visits, prior to the inclusion of their information in the study.

### 2.2. Data Extraction

Clinical and laboratory characteristics of all subjects included in this study were extracted from the patient’s charts and the hospital electronic database. If a patient was admitted more than once during the study period, only the information from the initial admission was analyzed.

**Figure 1:**
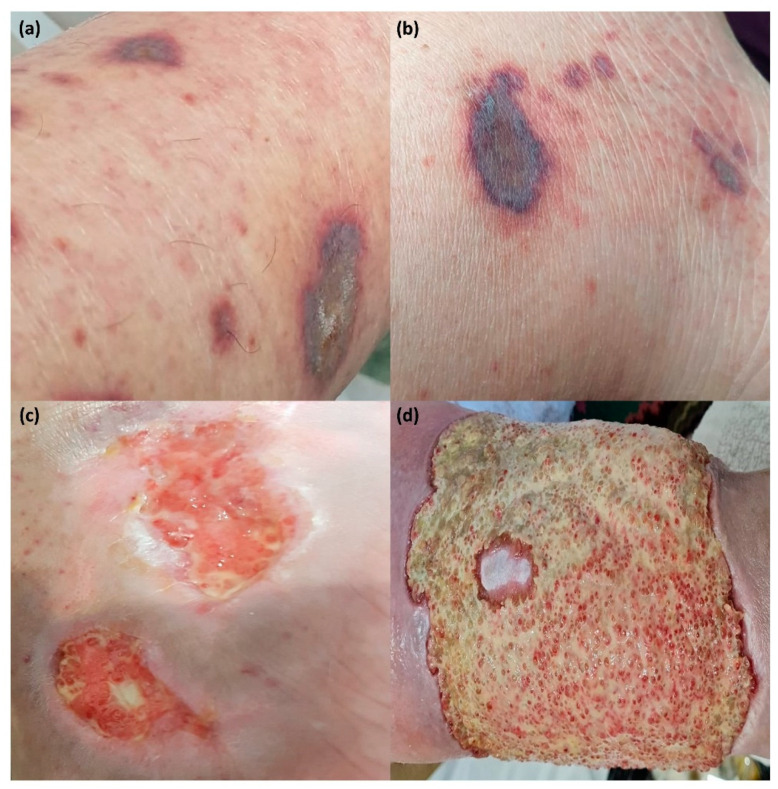
Distal lower limbs palpable purpura in a woman suffering from primary Sjögren’s Syndrome associated with leuko-cytoclastic vasculitis (**a**,**b**). In evolution, severe vasculitis manifestation and wide skin ulcer (**c**,**d**).

Data regarding patient’s cutaneous manifestations were registered throughout the study at the trimestral follow-up visits. Cutaneous manifestations such as: xeroderma, Raynaud syndrome and cutaneous vasculitis, including cryoglobulinemia vasculitis, leukocyto-clastic vasculitis and urticarial vasculitis, were recorded (Figure 1). Among laboratory markers, information was collected on complete blood count (CBC) and derived hematological ratios, namely NLR, PLR, MLR, as well as hs-C reactive protein (hs-CRP), complement levels (C3, C4), immuno-gram (IgA, IgM, IgG), rheumatoid factor (RF), proteins electrophoresis, serum cryoglobulins, antinuclear antibodies (ANA), anti-Ro/SSA and anti-La/SSB antibodies. Peripheral blood cells of the study subjects were collected in ethylenediaminetetraacetic acid (EDTA) tubes (Greiner Bio-One, Frickenhausen, Germany) and analyzed using Fluorescence Flow Cytometry technology by Sysmex XN-3000 (Sysmex Corporation, Kobe, Japan). Gamma-globulins were determined by migration in the electric field using Sebia Capillarys Sebia 3 OCTA analyzer (Sebia, Lisses, France). The immunological parameters were determined by immunoturbidimetry and analyzed on Beckman Coulter AU5812 (Beckman Coulter, Inc., Brea, CA, USA), while antibodies were determined by enzyme-linked immunosorbent assay (ELISA) technology on ORGENTEC Alegria 2 analyzer (ORGENTEC Diagnostika GmbH, Mainz, Germany).

### 2.3. Statistical Analysis

Statistical analysis was performed using SPSS software (version 26.0, SPSS, Chicago, IL, USA). The normality of distribution was checked by Kolmogorov–Smirnov test and Shapiro-Wilk test, and parametric or non-parametric tests were used on data according to normal or non-normal distributions. Continuous data were expressed as mean ± standard deviation while categorical data were presented as percentages. An unpaired Student’s *t*-test was used for comparing the difference between two groups when the continuous data fitted normal distribution. Mann-Whitney U test was used to compare the differences for non-parametric data between the groups. The Spearman correlation coefficient was computed to examine the association between two continuous variables and ROC curve analysis was performed to determine the sensitivity and specificity of the investigated parameters. Furthermore, the parameters which showed correlations in bivariate analysis were subsequently tested by linear regression analysis. Statistical significance was defined as *p* < 0.05.

## 3. Results

### 3.1. General Characteristics of the Study Groups

The demographical characteristics and laboratory findings of the study groups are presented in Table 1. In the pSS patients group, there were 121 female and 3 male participants with a mean age of 48.55 ± 10.45 (range: 19–68), while in the control group there were 119 female and two male participants with a mean age of 48.37 ± 8.92 (range: 20–67). The incidence of diagnosis in the pSS patients group was most frequently in the 4th and 5th decades of life, represented by 33 patients (26.6%) and 47 patients (37.9%), respectively. The age and gender composition were similar between the two groups (*p* = 0.887 and *p* = 0.671, respectively).

Lower values for erythrocytes, leukocytes, neutrophils, lymphocytes, monocytes and platelets were detected in the patients’ group compared to control group (*p* < 0.001). Immuno-gram parameters—IgA, IgG, IgM—were significantly higher in the patient’s group (*p* < 0.001). Gammaglobulins revealed similar differences, with a mean value of 1.45 ± 0.40 g/dL in pSS group, compared to 1.05 ± 0.16 g/dL in the control group, (*p* < 0.001). Likewise, RF was significantly higher in the patients group (*p* < 0.001). For the complement fractions, C3 was significantly higher in patients group (*p* < 0.003), while C4 was significantly lower in pSS patients compared to control group (*p* < 0.001). In the patients group, the value of hs-CRP was significantly higher, with a mean value of 24.22 ± 25.53 mg/L versus 5.75 ± 4.74 mg/L in the control group (*p* < 0.001).

Hematological ratios: MLR, PLR and NLR were significantly higher in the patients group. The mean value for MLR was 0.29 ± 0.12 in the patient group compared to 0.1 ± 0.52 in the control group (*p* < 0.001). For NLR, the mean value was 2.8 ± 0.71 in the patients group compared to 2.2 ± 0.96 in the control group (*p* < 0.001), while for PLR was 233.75 ± 56.34 in patients group compared to 153.7 ± 60.8 in the control group (*p* < 0.001). No statistically significant differences were detected for RDW-CV, PDWPCTR and MPVPCTR, between the two groups.

Of 124 patients, 105 (84.67%) were ANA and anti-Ro/SSA antibodies positive and 76 (61.29%) were anti-La/SSB antibodies positive. The mean VAS score was 18.87 ± 26.9 and the mean ESSDAI score was 16.96 ± 7.8 (range: 4–37). Most patients had with an ESSDAI score of moderate to severe disease (59.67%), while mild disease activity was detected in 50 patients (40.32%).

### 3.2. Dermatological Manifestations in Patients with pSS

Of 124 patients with pSS, 113 (91.12%) had xerostomia and 75 (60.48%) had xerophthalmia, the most common glandular manifestations. Cutaneous manifestations (CM) were present in 50 (40.32%) patients and no vasculitis lesions were present at the moment of diagnosis. During the follow up period, 34 patients (27.41%) had developed cutaneous vasculitis lesions and 66 patients (48.38%) non-vasculitis lesions. Cutaneous vasculitis lesions were represented by non–palpable purpura in 23 patients (18.54%), palpable purpura in 10 patients (8.06%) and by urticarial vasculitis in one patient (0.8%). Cryoglobulins were present in eight patients (6.45%) with palpable purpura. The cutaneous biopsy conducted for these eight patients with positive cryoglobulins, confirmed the diagnosis of leukocyto-clastic vasculitis in two cases (1.61%).

The most common non-vasculitis lesions were xeroderma, confirmed in 44 patients (35.48%), followed by Raynaud syndrome in 22 patients (17.74%). We mention that throughout the study several subjects with cutaneous manifestations presented, at the same time, vasculitis and non-vasculitis lesions (Table 2).

### 3.3. Analysis of Hematological Parameters in pSS Patients with Cutaneous Vasculitis Lesions

Further, bivariate analysis was conducted, based on the presence of cutaneous vasculitis in patients diagnosed with pSS, taking into consideration the hematological parameters determined at the moment of initial diagnosis. We found that circulating neutrophils and platelets were positively correlated with cutaneous vasculitis in pSS patients (*p* < 0.001), while for lymphocytes there was a negative correlation (*p* < 0.001). Furthermore, the cellular ratios revealed significant positive correlations with cutaneous vasculitis lesions. Thus, increased NLR values were correlated with the development of cutaneous vasculitis in pSS (r = 0.481, *p* < 0.001). Similar trends were detected for PLR (r = 0.524, *p* < 0.001) and MLR (r = 0.375, *p* < 0.001).

Analysis of immunological parameters revealed positive correlations between the level of gammaglobulins and CVL in pSS patients (r = 0.674, *p* < 0.001). As expected, there were positive correlations with cutaneous vasculitis for both ESSDAI and VAS scores (*p* < 0.001). There were no significant correlations with CVL for monocytes, MPWPCTR and PDWPCTR, C3 and C4, immunoglobulins, RF, hs-CRP, anti-Ro/SSA and Anti-La/SSB antibodies in pSS patients (Table 3).

### 3.4. Receiver-Operating Characteristic (ROC) Curves of NLR, PLR, MLR and Gammaglobulins for the Prediction of Cutaneous Vasculitis in pSS Patients

**Figure 2:**
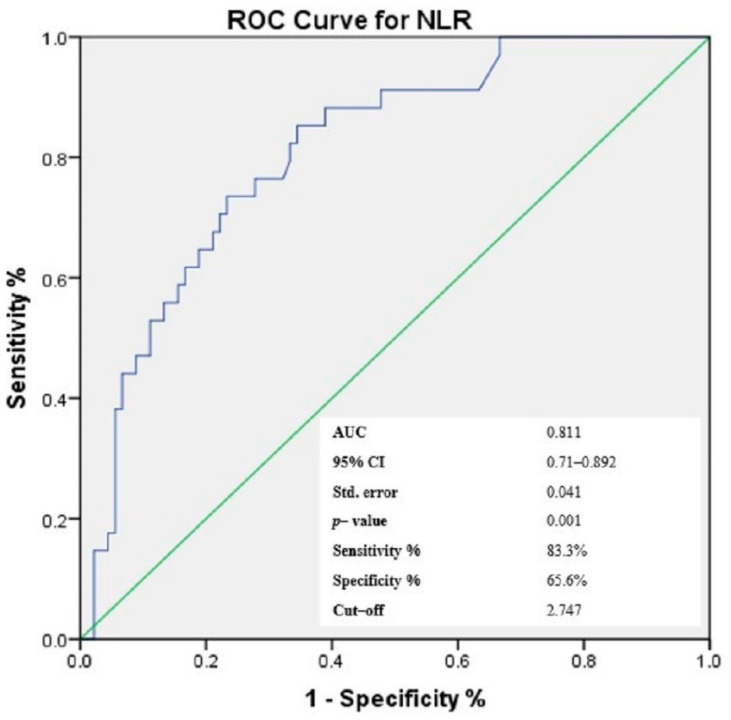
Receiver operating characteristic curve of NLR levels for the prediction of the cutaneous vasculitis lesions in patients with pSS.

**Figure 3:**
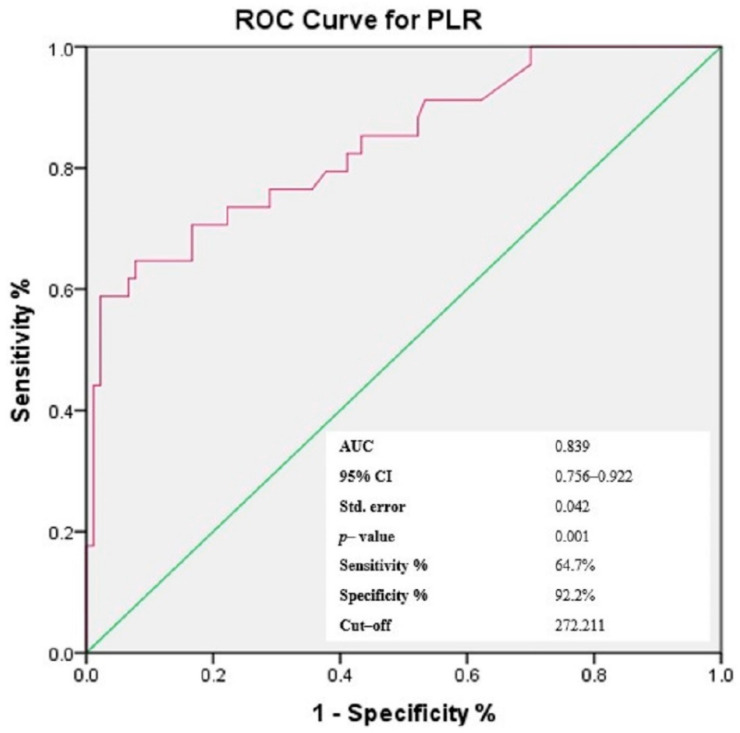
Receiver operating characteristic curve of PLR levels for the prediction of the cutaneous vasculitis lesions in patients with pSS.

**Figure 4:**
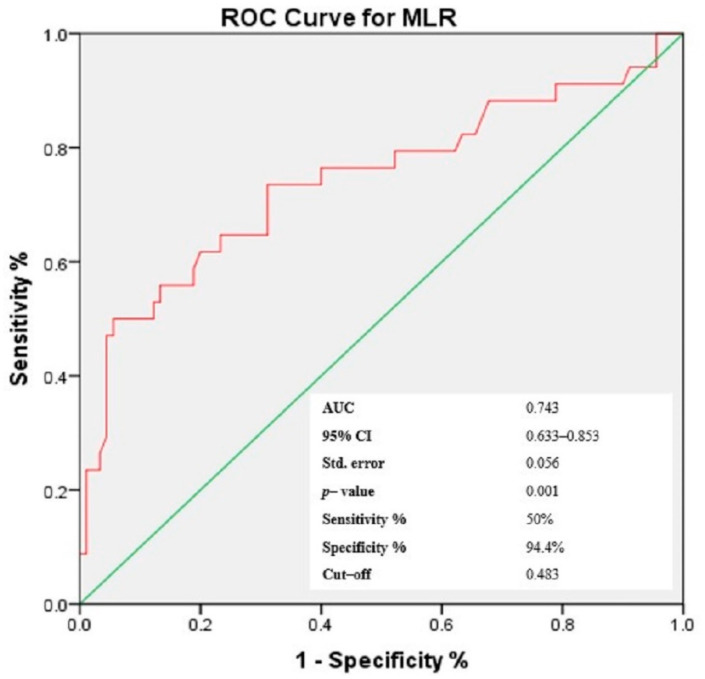
Receiver operating characteristic curve of MLR levels for the prediction of the cutaneous vasculitis lesions in patients with pSS.

**Figure 5:**
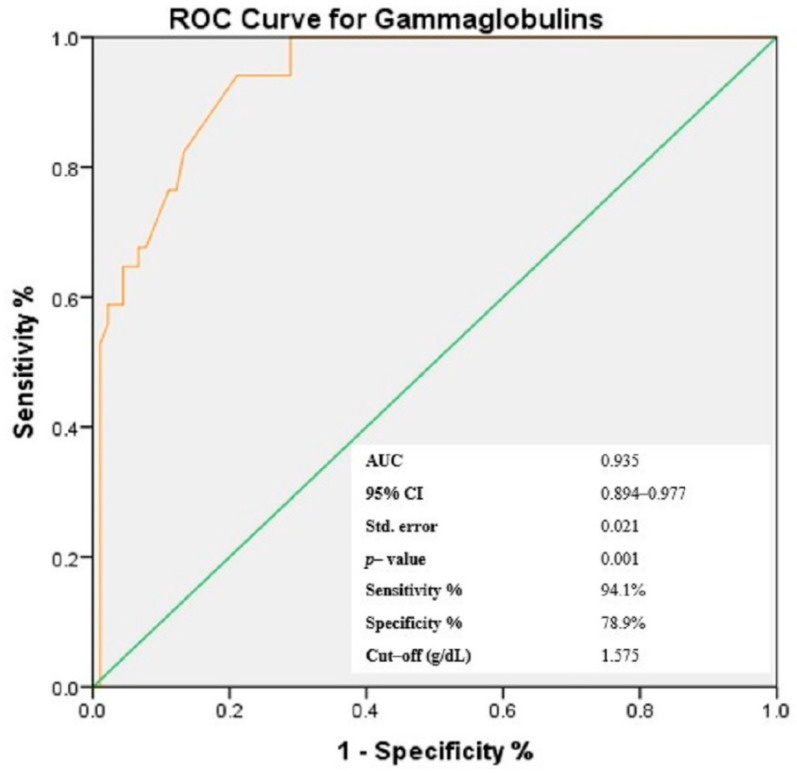
Receiver operating characteristic curve of gammaglobulins levels for the prediction of the cutaneous vasculitis lesions in patients with pSS.

Based on the findings presented above and taking into consideration that the aim of the study was to identify easily accessible prediction elements for cutaneous vasculitis lesions in patients with pSS, we have chosen to continue the analysis with a focus on cellular ratios—NLR, PLR and MLR, as elements that carry dual information from the types of circulating cells enclosed in each ratio, within one numerical value. Similarly, considering the significant correlation in bivariate analysis, gammaglobulins were also included for further investigation. Using the ROC analysis, the optimal threshold for the three ratios and gammaglobulins was determined, maximizing the composite of specificity and sensitivity for the prediction of cutaneous vasculitis lesions in pSS patients (Figure 2, Figure 3, Figure 4 and Figure 5). In predicting the development of CVL in pSS patients, the area under the curve (AUC) for NLR was 0.811 (*p* < 0.001), with a sensitivity of 85.3% and a specificity of 65.6%. For PLR, ROC curve analysis resulted in an AUC of 0.839 (*p* < 0.001), with a sensitivity of 64.7% and a specificity of 92.2%, while for MLR, the AUC area was 0.743 (*p* < 0.001), with a sensitivity of 50% and a specificity of 94.4%. In addition, for gammaglobulins the AUC was 0.935 (*p* < 0.001), with a sensitivity of 94.1% and a specificity of 78.9%. Based on the ROC curve analysis, the optimal cut-off values for the cellular ratios were: NLR = 2.747, PLR = 272.211 and MLR = 0.483, while for gammaglobulins the optimal cut-off value was 1.575 g/dL.

The multivariate analysis, conducted using multiple linear regression, considered for the analysis hematological parameters, immunological parameters and the age of the patients as elements that might influence the development of CVL in pSS. We did not include gender of the patients in this analysis due to the discrepancy in male to female ratio (three males and 121 females). The ESSDAI and VAS scales were excluded from this analysis as they include within the scale the dependable variable. The independent prediction character for the development of cutaneous vasculitis lesions was confirmed for all three hematological ratios investigated in the study: NLR (95% CI 0.053 to 0.231, *p* < 0.002), PLR (95% CI 0.001 to 0.003, *p* < 0.003) and MLR (95% CI 0.086 to 0935, *p* < 0.019). Anti-La/SSB and anti-Ro/SSA antibodies did not reach the threshold of statistical significance in the multivariate analysis (*p* < 0.937 respectively, *p* < 0.215). In addition, gammaglobulins have proven an independent prognostic character for the development of CVL (95% CI 0.423 to 0.688, *p* < 0.001) (Table 4). The R square for this model was 0.645 (*p* = 0.001).

## 4. Discussion

Primary Sjögren’s Syndrome (pSS) is not a unique entity but a syndrome with different clinical phenotypes that can involve glandular and extra-glandular manifestations. Many simple or complex molecules, sometimes costly and technologically challenging, have been investigated over time as potential biomarkers in immune diseases, including pSS. However, studies related to the usefulness of globally available and inexpensive tests to assess the severity and the risk for extra-glandular manifestations in pSS are still lacking. In this study, we have investigated the prognostic potential of different hematological parameters, determined routinely in pSS, for the development of cutaneous vasculitis lesions (CVL). These manifestations, even when diagnosed as single, transient episodes were reported to be associated with increased severity of the disease and multiple extra-glandular involvement [22]. Furthermore, recent reports suggest a link between CVL and the occurrence of lymphoma in patients with pSS, emphasizing once more the importance of the early detection of high-risk patients for CVL in pSS [23,24].

The analysis of the study groups revealed that routine blood tests show major differences in pSS compared to control subjects. Immunological and inflammatory parameters were significantly increased, while circulating cellular elements were decreased considerably in pSS patients. Serum components, traditionally used in the diagnosis and monitoring of pSS and other autoimmune diseases: RF, immunoglobulins, and gamma-globulins, were intensely expressed in pSS patients, in accordance with pre-existing studies [12,13,25]. The prevalence of autoantibodies, namely ANA, anti-La/SSB and anti-Ro/SSA in our group of patients was comparable to previously reported data [12,25,26]. We found a significantly reduced lymphocyte count in peripheral blood of pSS patients. An intense tissue infiltration with lymphocytes has been detected in autoimmune diseases, also associated with increased rates of lymphocyte apoptosis [18]. These cells are key players in autoimmune diseases and their presence was confirmed in all tissues affected by chronic inflammation. Furthermore, in pSS, lymphocytes participate in the intratissue production of autoantibodies and might be associated with the risk of developing lymphoma due to chronic antigenic stimulation [27]. Alongside leukopenia, anemia and thrombocytopenia were the most frequent cellular anomalies reported in pSS [28]. Similar trends were confirmed in our group of patients, in whom the mean counts for erythrocytes and platelets were significantly lower compared to healthy controls, even though their numbers fell within the normal ranges for these parameters. Furthermore, the analysis of cellular ratios—NLR, MLR and PLR—revealed higher values in pSS patients compared to controls, suggesting that, even though all cellular lines were decreased in pSS patients, the important drop in circulating lymphocytes counts is the main actor to influence these differences. These findings are supported by similar results reported from previous studies investigating the hematological profile of pSS patients [13,18,29]. Disease activity, quantified through ESSDAI and VAS scores, was higher in our group of pSS patients compared to previous studies [13,21], probably due to a late diagnosis of the disease. It is not uncommon for many patients to seek medical assistance a long time after the onset of sicca syndrome, sometimes only after the emergence of extra-glandular complications in pSS, leading to a significant delay in diagnosing this disease and increased rates of mortality [30].

The epidemiology of our pSS patients highlighted a female predominance and respected the two peaks of incidence of the disease in the 4th and 5th decades of life, in accordance with previous reports [12,26]. The diagnostic criteria for pSS—xerostomia and xerophthalmia—were confirmed in the majority of patients, while extra-glandular cutaneous manifestations were detected in almost half of the patients throughout the study. Similar findings were reported in previous studies [31]. Cutaneous involvement, the most frequent extra-glandular manifestation in pSS, can be expressed through a variety of clinical aspects, ranging from common non-vasculitis lesions, such as xeroderma, to extremely rare events, such as urticarial vasculitis [5]. In our group of patients, the distribution of cutaneous vasculitis and non-vasculitis lesions subtypes showed trends comparable to previously reported data [26,32,33].

Even if numerous efforts have been made to identify the mechanisms involved, the pathophysiology of cutaneous vasculitis in pSS is not completely understood. Histopathology specimens from patients with cutaneous vasculitis lesions revealed different patterns of tissue alterations. In leuko-cytoclastic vasculitis, an intense infiltration with neutrophils was described, with extensive necrosis of the cutaneous vascular walls. In other cases, the vascular walls were intact, while effectors on the chronic autoimmune response—lymphocytes, histiocytes and plasma cells—were predominant elements in the tissue infiltrate [34]. Small skin vessels were almost exclusively involved in these changes, with only sporadic reports on the involvement of medium size vessels [33]. One hypothesis is that neutrophilic and lymphocytic vasculitis detected in pSS skin specimens are actually progressive stages of the same autoimmune process [33]. It is considered that antibodies against anti-Ro/SSA and anti-La/SSB antigens create immune complexes which precipitate and affect walls of the blood vessels [35]. CVL in pSS were associated with laboratory findings, such as the presence of anti-Ro/SSA and anti-La/SSB antibodies, monoclonal gammopathy, cryoglobulinemia and hypocomplementemia [22,32,36]. Previous research conducted on salivary tissue specimens from pSS patients revealed a strong positive correlation between the circulating titers of antibodies and their presence in the glandular tissue specimens [37]. Based on these reports, similar changes in skin small vessel walls, with increased tissue expression of anti-Ro and anti-La antibodies, could lead to the clinical manifestations of cutaneous vasculitis in pSS patients. Other immunological markers, such as gamma-globulins, showed strong independent correlation with the emergence of CVL in our group of patients. Previous studies report hypergammaglobulinemia as a common finding in pSS patients with cutaneous vasculitis lesions [38]. These changes are related to the activation and proliferation of B cell populations, which lead to lymphomatous escape in the target tissues in pSS, namely salivary and lacrimal glands [39]. Furthermore, in skin biopsies collected from pSS patients with cutaneous manifestations, B-cell aggregates were frequently found, suggesting their important role in the pathogenesis of pSS and the development of extra-glandular manifestations, such as CVL [40]. These pathophysiological mechanisms might also explain the occurrence of B cell lymphoma in pSS patients with CVL [23].

In our group of pSS patients, the investigation of the predictive potential for the development of CVL enclosed in hematological elements revealed a positive correlation with the platelet and neutrophil counts determined at the moment of disease diagnosis. Similarly, a negative correlation was detected between the emergence of CVL and the lymphocyte count. Furthermore, the bivariate analysis revealed that all cellular ratios—NLR, PLR and MLR—had a strong positive correlation with the development of CVL in pSS patients. The subsequent multivariate analysis confirmed the independent predictive character for all of three investigated cellular ratios: NLR, PLR and MLR. To our knowledge this is the first study to identify and report these findings, which link these standard hematological parameters, routinely determined in the general assessment of all pSS patients with the development of CVL.

Skin vasculitis manifestations have been associated with increased disease activity in pSS [21]. In our group of patients, ESSDAI and VAS scores revealed positive correlations with CVL, confirming once more that these types of manifestations are more likely to occur in patients with increased disease activity. Previous reports have suggested an independent association between NLR and disease activity, assessed through the ESSDAI score in pSS patients [12]. Similar findings were reported in other autoimmune diseases, connecting hematological elements with disease severity. Thus, in SLE, positive correlations were found between hematological parameters NLR and PLR with the disease activity score, which also correlated negatively with lymphocyte count [15]. Likewise, in rheumatoid arthritis, Behçet disease, Takayasu arteritis and systemic sclerosis, positive correlations were found between NLR, PLR and MLR and the disease activity scores [41,42,43,44]. In Behçet disease, NLR was closely related with the development of skin manifestations [42]. Other reports suggest an association between MLR and NLR, and vascular and cutaneous manifestations in systemic sclerosis [44]. The superior predictive power of NLR, PLR and MLR compared to their individual components is due to the discrete variability in their individual components. These differences become more obvious when analyzed together in a unique parameter that collects the information from each of its individual components. Currently, the diagnosis of these manifestations is based on the interdisciplinary collaboration between rheumatologists, dermatologists and internal medicine specialists. In our opinion, easily detectable serum elements, which could place the patients into high risk groups for the emergence of CVL, might prove to be very useful for an early detection of these types of extra-glandular manifestations in pSS, leading to a timely and more efficient treatment, using individualized therapeutic strategies for the benefit of the patients. Furthermore, previously reported higher mortality rates and prolonged hospitalization associated with CVL in pSS are a strong arguments for the need to identify predictive elements for the development of these manifestations [34]. Even when the skin involvement is not severe, it can impair a patient’s quality of life and significantly increase morbidity and the associated financial burden for the healthcare system.

This study has several limitations related to its retrospective, single center character and the relatively small sample size, however comparable to previous reports. Furthermore, even if they were not within the scope of this study, the analysis of the time interval from diagnosis of pSS until CVL occurrence, as well as the influence of pSS specific therapies on CVL occurrence are aspects that might provide additional valuable information on disease progression, complications and response to treatment. Therefore, additional prospective studies, conducted in multiple centers and considering large groups of patients are necessary in order to validate our findings.

## 5. Conclusions

Our findings suggest a strong connection between classical biological parameters, determined routinely to follow the pSS evolution and the subsequent development of CVL. NLR, PLR and MLR were increased in pSS patients, positively correlated with CVL and confirmed their independent predictive character for the development of CVL. Additionally, gammaglobulins confirmed their independent prediction character in CVL development. Hence, our results support that these cost-effective and widely available parameters could become valid elements that might be used for the early detection of patients at risk for the development of CVL.

## Figures and Tables

**Table 1:** Demographic and laboratory results of patients and control group.

Demographic Characteristics	Patients Group No = 124	Control Group No = 121	*p*-Value
	Mean ± SD/Median	Mean ± SD/Median	
Age (years)	48.55 ± 10.45	48.37 ± 8.92	0.887 ^b^
Sex Female (*n*, %) Male (*n*, %)	121 (97.6%)	119 (98.3%)	0.671 ^c^
	3 (2.4%)	2 (1.7%)	
**Laboratory findings Cell counts**			
Erythrocytes (×10^6^/µL)	4.22 ± 0.38	4.44 ± 0.38	0.001 ^a^
Leucocytes (10^9^/µL)	5.1 ± 1.71	6.94 ± 2.17	0.001 ^a^
Neutrophils (10^9^/µL)	3.11 ± 0.67	4.27 ± 1.96	0.001 ^a^
Lymphocytes (10^9^/µL)	1.12 ± 0.09	1.97 ± 1.21	0.001 ^a^
Monocyte (10^9^/µL)	0.33 ± 0.14	0.64 ± 0.16	0.001 ^a^
Platelets (10^9^/L)	25.8 ± 51.4	29.6 ± 68.18	0.001 ^a^
RDW-CV (%)	12.97 ± 1.16	13.13 ± 0.84	0.086 ^a^
**Cellular ratios**			
MPVPCTR	39.33 ± 10.93	42.22 ± 25.36	0.246 ^b^
PDWPCTR	46.46 ± 12.7	49.76 ± 29.45	0.255 ^b^
MLR	0.29 ± 0.12	0.1 ± 0.52	0.001 ^a^
NLR	2.8 ± 0.71	2.2 ± 0.96	0.001 ^a^
PLR	233.75 ± 56.34	153.7 ± 60.8	0.001 ^a^
**Immunological results**			
RF (U/mL)	52.18 ± 61	9.32 ± 18.55	0.001 ^a^
IgA (mg/dL)	293.67 ± 15.98	222.9 ± 93.9	0.001 ^a^
IgG (mg/dL)	1656.94 ± 708.73	1200.5 ± 315.2	0.001 ^a^
IgM (mg/dL)	168.4 ± 121.31	116.84 ± 45.75	0.001 ^a^
Gammaglobulins (g/dL)	1.45 ± 0.4	1.05 ± 0.16	0.001 ^a^
C3 (mg/dL)	133.48 ± 116.22	131.33 ± 16.27	0.003 ^a^
C4 (mg/dL)	22.02 ± 10.33	32.32 ± 10.7	0.001 ^a^
ANA Positive (*n*, %) Negative (*n*, %)	105 (84.67%)	-	-
	19 (15.32%)	-	-
Anti-Ro/SSA Positive (*n*, %) Negative (*n*, %)	105 (84.67%)	-	-
	19 (15.32%)	-	-
Anti-La/SSB Positive (*n*, %) Negative (*n*, %)	76 (61.29%)	-	-
	48 (38.70%)	-	-
**Inflammatory results**			
hs-CRP (mg/L)	24.22 ± 25.53	5.75 ± 4.74	0.001 ^a^
**Clinical scores**			
ESSDAI score	16.96 ± 7.8	-	-
ESSDAI score 5–13	50 (40.32%)	-	-
ESSDAI score ≥14	74 (59.67%)	-	-
VAS pain score (mm)	18.87 ± 26.9	-	-

Abbreviations: RDW-CV, red cell distribution width-coefficient of variation; MPVPCTR, mean platelet volume to Plateletcrit ratio; PDWPCTR, platelet distribution width to platelet crit ratio; MLR, monocyte to lymphocyte ratio; NLR, neutrophil to lymphocyte ratio; PLR, platelet to lymphocyte ratio; RF, rheumatoid factor; C3 and C4, complement 3 and 4; hs-CRP, high sensitivity C-reactive protein; ANA, antinuclear antibodies; SD, standard deviation; VAS, visual analogue scale; ESSDAI, EULAR Sjögren’s Syndrome Disease Activity Index. ^a^ Mann–Whitney U test, ^b^ Independent samples *t*-test, ^c^ Pearson Chi-Square; statistical significance < 0.05.

**Table 2:** Cutaneous manifestations in pSS patients.

Clinical Manifestations	Number of Patients (%)
Xerostomia (*n*, %)	113 (91.12%)
Xerophthalmia (*n*, %)	75 (60.48%)
CM (*n*, %)	50 (40.32%)
Cutaneous vasculitis lesions (*n*, %)	34 (27.41%)
Non–palpable purpura	23 (18.54%)
Palpable purpura	10 (8.06%)
Cryoglobulinemic vasculitis (*n*, %)	8 (6.45%)
Leukocytoclastic vasculitis (*n*, %)	2 (1.61%)
Urticarial vasculitis (*n*, %)	1 (0.8%)
Non vasculitis lesions	66 (48.38%)
Xeroderma (*n*, %)	44 (35.48%)
Raynaud syndrome (*n*, %)	22 (17.74%)

**Table 3:** Correlation between hematological parameters and cutaneous vasculitis lesions.

	Cutaneous Vasculitis Lesions	
	Correlation Coefficient (r)	*p*-Value
Lymphocytes (10^9^/µL)	−0.432	0.001 *
Neutrophils (10^9^/µL)	0.389	0.001 *
Monocytes (10^9^/L)	−0.150	0.097
Platelets (10^9^/L)	0.467	0.001 *
NLR	0.481	0.001 *
PLR	0.524	0.001 *
MLR	0.375	0.001 *
MPWPCTR	−0.080	0.380
PDWPCTR	−0.029	0.753
C3 (mg/dL)	−0.101	0.267
C4 (mg/dL)	−0.123	0.174
IgA (mg/dL)	−0.067	0.457
IgG (mg/dL)	−0.168	0.063
IgM (mg/dL)	0.057	0.531
Gammaglobulins g/dL)	0.674	0.001 *
RF (U/mL)	0.176	0.051
hs-CRP (mg/L)	0.077	0.398
Anti-Ro/SSA (U/mL)	0.211	0.280
Anti-La/SSB (U/mL)	0.414	0.791
VAS pain score (mm)	0.571	0.001 *
ESSDAI score	0.444	0.001 *

Abbreviation: MPVPCTR, mean platelet volume to Plateletcrit ratio; PDWPCTR, platelet distribution width to platelet crit ratio; MLR, monocyte to lymphocyte ratio; NLR, neutrophil to lymphocyte ratio; PLR, platelet to lymphocyte ratio; RF, rheumatoid factor; C3 and C4, complement 3 and 4; hs-CRP, high sensitivity C-reactive protein; ANA, antinuclear antibodies; SD, standard deviation; VAS, visual analogue scale; ESSDAI, EULAR Sjögren’s Syndrome Disease Activity Index. Bold values indicate statistical significance (* *p* < 0.05). Data were analyzed using the Spearman approach.

**Table 4:** Multiple linear regression analysis for the development of cutaneous vasculitis lesions in pSS.

Laboratory Findings	Unstandardized Coefficients		Standardized Coefficients	T	*p*	95.0% Confidence Interval for B	
	Β	Standard Error	Beta			Lower Bound	Upper Bound
(Constant)	−1.585	0.176	−	−9.012	0.000	−1.934	−1.237
NLR	0.142	0.045	0.226	3.156	0.002 *	0.053	0.2
PLR	0.002	0.001	0.230	3.064	0.003 *	0.001	0.003
MLR	0.510	0.214	0.147	2.380	0.019 *	0.086	0.935
Gammaglobulins (g/dL)	0.555	0.067	0.499	8.291	0.001 *	0.423	0.688
Anti-Ro/SSA	0.000	0.000	0.074	1.247	0.215	0.000	0.001
Anti-La/SSB	−2.15 × 10^−5^	0.000	−0.005	−0.079	0.937	−0.001	0.000
Age	0.000	0.002	0.009	0.154	0.878	−0.004	0.005

Abbreviations: MLR, monocyte to lymphocyte ratio; NLR, neutrophil to lymphocyte ratio; PLR, platelet to lymphocyte ratio; B, unstandardized coefficient representing the regression slope; T, coefficient divided by the standard error. Bold values indicate statistical significance (* *p* < 0.05).

## Data Availability

The datasets used and/or analyzed during the present study are available from the corresponding author.
